# Protective effect of the aqueous extract of *Tabernaemontana stapfiana* (Apocynaceae) stem bark against monosodium glutamate-induced neurotoxicity in Swiss albino mice

**DOI:** 10.1016/j.ibneur.2026.04.004

**Published:** 2026-04-22

**Authors:** Kada Sanda Antoine, Kouémou Nadège Emégam, Mboloh Patrick, Oumar Mahamat, Mumbi Laurantine Ngenteh, Salah Anchang Martin

**Affiliations:** a Department of Zoology, Faculty of Science, University of Bamenda, Cameroon P.O. Box 39, Bambili, Cameroon; b Department of Animal Biology and Conservation, Faculty of Science, University of Buea, Cameroon P.O. Box 63, Buea, Cameroon; c Department of Biology, Higher Teacher Training College, University of Bamenda, P.O. Box 39 Bambili, Cameroon

**Keywords:** Monosodium glutamate, Neurodegeneration, *Tabernaemontana stapfiana*, Oxidative stress, Antioxidants, Acetylcholinesterase

## Abstract

**Background:**

Monosodium glutamate (MSG), a common food additive, is known to enhance taste and flavour. However, increasing evidence suggests that elevated MSG intake is linked to neuronal pathologies and death. *Tabernaemontana stapfiana is a plant traditionally used in Cameroon for multiple purposes, including as a haemostatic, an anthelmintic, and in the treatment of rheumatism, kidney disorders, rickets, and conjunctivitis. Notably, traditional practitioners also use it to address nervous system disorders, drug addiction, and memory impairments, highlighting its potential as a neuroprotective agent. This study aimed to evaluate the neuroprotective effects of the aqueous extract of the stem barks from this plant on MSG-induced neurotoxicity in mice.*

**Methods:**

This study was carried out using 49 Swiss albino mice, classified into seven groups of 7 mice each. The negative control group (group 1) received distilled water (10 mL/kg) throughout this experiment. The positive control group received MSG (2 g/kg) and distilled water (10 mL/kg). Reference standard 1 (RS1) received vitamin C (100 mg/kg) and reference standard 2 (RS2) received donepezil (3 mg/kg). Doses 1, 2, and 3 groups received MSG concomitantly with aqueous extract of *T. stapfiana* (250, 500, and 1000 mg/kg respectively) for 10 days. Monosodium glutamate was administered intraperitoneal (*i.p*) while treatments were orally administered (*p.o*). Behavioural parameters such as Y-maze, elevated plus maze, open field and wire suspension tests were evaluated on days 8, 9, and 10 using various tests. After 10 days, biomarkers of oxidative stress and acetylcholinesterase activity were evaluated in the brain.

**Results:**

MSG induced oxidative stress, poor muscle coordination, and spatial short-term memory impairment in mice. The *T. stapfiana extract prevented these MSG-induced behavioural changes. The extract also alleviated oxidative stress and reduced acetylcholinesterase activity in the brains of the animals.*

**Conclusion:**

Results suggest that *T. stapfiana aqueous extract prevents neurotoxicity, possibly through its free radical scavenging ability and the maintenance of acetylcholinesterase activity in a mouse model of MSG-induced neurotoxicity.*

## Graphical Abstract

**Figure:**
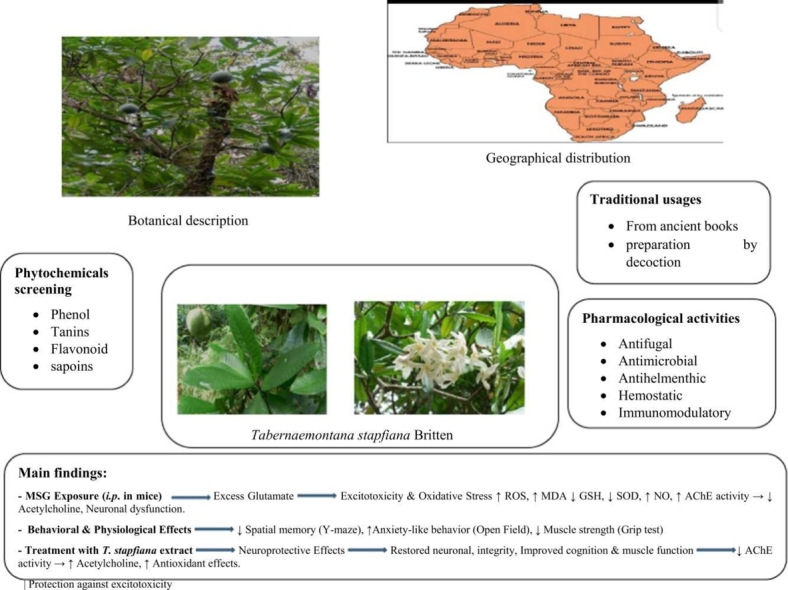

## 1 Introduction

Monosodium glutamate (MSG) is the sodium salt of glutamic acid and is widely used as a flavour enhancer due to its ability to impart the umami taste, thereby improving food palatability. Ajinomoto® (a commercial formulation of MSG produced by Ajinomoto Co., Inc.) is commonly used in many regions (Mortensen et al., 2017). International regulatory authorities, including the World Health Organization / Food and Agriculture Organization Joint Expert Committee on Food Additives and the European Food Safety Authority, have concluded that MSG is safe for consumption at typical dietary levels, with EFSA establishing an acceptable daily intake of 30 mg/kg body weight/day for glutamates (Mortensen et al., 2017). Chemically, MSG has the formula C₅H₈NO₄Na, a molar mass of 187.12 g/mol, and a melting point of approximately 232°C (Al-Otaibi et al., 2022). Beyond its culinary applications, glutamate the active component of MSG functions as a major excitatory neurotransmitter in the central nervous system and plays a critical role in learning and memory processes. However, excessive exposure to MSG has been associated with neurotoxic effects. At high systemic doses, MSG may induce neuronal damage through glutamate-mediated excitotoxicity, oxidative stress, and disruption of neurotransmitter balance. The neurotoxicity induced by MSG is primarily attributed to excitotoxicity, a process in which excessive glutamate overstimulates neuronal receptors, leading to calcium ion (Ca²⁺) influx, mitochondrial dysfunction, and increased production of reactive oxygen species. Elevated glutamate levels can overstimulate NMDA and AMPA receptors, resulting in impaired neurotransmission and neuronal injury (Subramanian et al., 2023). Due to these effects, MSG-induced neurotoxicity is widely used as an experimental model to investigate mechanisms of neuronal damage and to evaluate potential neuroprotective agents (Behera, 2020, Piniella and Zafra, 2023). Such neurotoxic mechanisms have been implicated in several neurological disorders, including Alzheimer’s disease, Parkinson’s disease, and epilepsy. In particular, Alzheimer’s and Parkinson’s diseases are progressive, age-related neurodegenerative conditions characterized by cognitive decline and memory impairment. These disorders are highly debilitating and their global prevalence is increasing due to rising life expectancy. Current pharmacological treatments are largely symptomatic and do not effectively halt disease progression, while access to such therapies remains limited in many rural and low-resource settings due to high costs and availability constraints (Behera, 2020). These challenges underscore the need to explore alternative therapeutic strategies, particularly those derived from natural sources. Medicinal plants have historically been an important source of bioactive compounds with neuroprotective potential. *Tabernaemontana stapfiana*, a plant traditionally used in Cameroon, exhibits a wide range of medicinal properties. It has been employed as an anaesthetic, anthelmintic, and in the management of memory impairments, rheumatism, kidney disorders, rickets, and conjunctivitis (Kuete et al., 2010). Notably, its traditional application in management of nervous system disorders and improving memory suggests that *T. stapfiana* may possess compounds capable of modulating neuronal function or protecting against neurotoxicity.

Given this background, the present study was designed to evaluate the neuroprotective potential of the aqueous extract of *T. stapfiana* stem bark against MSG-induced neurotoxicity in mice. Specifically, the study investigates the effects of the extract on behavioural outcomes, oxidative stress biomarkers, and acetylcholinesterase activity. MSG was employed in this study as an experimental excitotoxin to induce neurotoxicity. Through these analyses, we aim to provide scientific validation for the traditional use of *T. stapfiana* and explore its potential as a natural therapeutic agent against MSG-related neurological damage.

## 2 Materials and methods

### 2.1 Chemicals

Monosodium glutamate (MSG) was obtained from Alfa Aesar (Germany). Ethanol was purchased from EK Industries Inc. (USA) as a reagent‑grade solvent. Donepezil hydrochloride was sourced from FUJIFILM Wako Pure Chemical Corporation (Japan). Vitamin C (ascorbic acid) tablets were sourced from AdvaCare Pharma (via their AdvaLife™/Adva‑C product line). Other chemicals including acetyl thiocholine iodide (ACTI), s‑butyrylthiocholine iodide (BCTI), 5,5′‑dithiobis (2‑nitrobenzoic acid) (DTNB), and trichloroacetic acid (TCA) were obtained from Sigma‑Aldrich (St. Louis, MO, USA). Thiobarbituric acid (TBA) was purchased from Griffin & George (Wembley, Middlesex, England). All chemicals were of analytical grade and used without further purification.

### 2.2 Plant material

The stem bark of *Tabernaemontana stapfiana* was collected in December 2023 from Wabane, South-West Region of Cameroon (GPS coordinates: 5.68299° N, 9.98448° E), where average daily temperatures range from 18°C to 28°C (64°F–82 °F) and relative humidity is typically between 70% and 85% during this period. The plant was authenticated at the National Herbarium of Cameroon by comparison with the reference specimen of Thomas D.W. 2621, and a voucher specimen was deposited under No. 50330 SRF/Cam. The collected stem bark was washed and air-dried at ambient temperature for seven days. The stem barks were cleaned and shade-dried for one week and ground into a fine powder using a grinding machine. The aqueous extract of *T. stapfiana* stem bark was prepared following a modified version of the method described by Kuete et al., 2010. A total of 240 g of the plant powder was boiled in 1.5 litres of distilled water for 30 min, filtered, and the resulting extract was evaporated in an oven at 45ºC for one week. The yield of 17.35% was obtained, and the extract stored for future use.

### 2.3 Laboratory animals

A total of 49 Swiss albino mice (both sexes = 25 females and 24 males) of two months old, weighing between 25 and 30 g, were obtained from the animal house of the University of Buea, Cameroon. The mice were housed in groups of seven per cage under controlled environmental conditions (room temperature, 40–60% humidity, and 12-hours light/ 12-hours dark cycle) and acclimatized for two weeks prior to monosodium glutamate (MSG) administration and treatments. The use of animals in this study was authorized by the Cameroon National Veterinary Laboratory (approval and health control reference No. 0498/25 CCS/MINEPIA/RD-NW/DDME/SSV). All animal experiments were conducted in compliance with the ARRIVE (Animal Research: Reporting of in Vivo Experiments) guidelines and carried out in accordance with the Guidance on the Operation of the Animals (Scientific Procedures) Act 1986 and associated guidelines, the EU Directive 2010/63/EU for the protection of animals used for scientific purposes, and the National Research Council Guide for the Care and Use of Laboratory Animals (NIH Publication No. 85–23, revised 1996). Both male and female animals were used in this study, and the potential influence of sex on the study outcomes was considered during data analysis.

### 2.4 Experimental design

A total of 49 Swiss Albino mice were used in this study. The sample size of n = 7 per group was chosen based on previous studies that have investigated the neuroprotective effects of natural products in monosodium glutamate-induced neurotoxicity in mice (Emégam et al., 2017, Nishigaki et al., 2018, Brant et al., 2023).

Mice were randomly assigned to one of seven treatment groups (n = 7 per group) using a random number generator in Microsoft excel. The investigators performing the behavioural tests and analysing the data were blinded to the treatment groups. Treatment solutions were prepared and coded by a researcher not involved in the behavioural testing or data analysis, and the code was not broken until after the data analysis was completed.

• Group 1 received distilled water (10 mL/kg), serving as the negative control,
• Group 2 received MSG (2 g/kg) + distilled water (10 mL/kg), serving as the positive control,
• Group 3 received vitamin C (100 mg/kg) + MSG (2 g/kg), serving as reference standard 1
• Group 4 received donepezil (3 mg/kg) + MSG (2 g/kg) serving as reference standard 2
• Group 5 received extract (250 mg/kg) + MSG (2 g/kg) serving as dose 1
• Group 6 received extract (500 mg/kg) + MSG (2 g/kg) serving as dose 2
• Group 7 received extract (1000 mg/kg) + MSG (2 g/kg) serving as dose 3

Vitamin C, was used as a reference antioxidant to evaluate the role of oxidative stress in MSG-induced neurotoxicity. Donepezil, a cholinesterase inhibitor was included as an additional reference to assess its potential to enhance cognitive function in this model it was used as a reference standard due to its established neuroprotective and memory-enhancing effects, and it has previously been applied in monosodium glutamate (MSG)-induced neurotoxicity models (Emégam et al., 2017). Monosodium glutamate (MSG) was administered intraperitoneally (*ip*) at a dose of 2 g/kg body weight, as described in previous studies that established this regimen as an experimental model for MSG-induced neurotoxicity (Carricondo et al., 2002, Emégam et al., 2017, Prastiwi et al., 2015, Subramanian et al., 2023). The intraperitoneal route was chosen to ensure reproducible systemic exposure and to reliably induce neurotoxic effects under controlled experimental conditions. Vitamin C was given orally (*po*) via gavage at 100 mg/kg, following the protocol described by Taiwo et al., 2024, while donepezil was administered orally via gavage at the dose of 3 mg/kg (Akinsehinwa et al., 2025). The plant extract was equally po and the doses of *Tabernaemontana stapfiana* extracts used in this study were selected based on our previous findings (Ngenteh et al., 2025), which demonstrated significant neuroprotective, antioxidant, and anti-inflammatory effects at these concentrations in Wistar rats. In addition, toxicological evaluations of related Tabernaemontana species have shown a high safety margin, with an LD₅₀ of approximately 6.75 g/kg and no significant toxicity observed at repeated doses up to 500 mg/kg (Kuete et al., 2010). Thus, the selected doses were considered both safe and biologically effective for the present investigation. Distilled water was administered orally at a dose of 10 mL/kg. Treatments were conducted once daily, with MSG given first, followed one hour later by the respective administrations: distilled water for Group 2, extract for Groups 5–7, vitamin C for Group 3, and donepezil for Group 4. This regimen was continued consecutively for 10 days.

### 2.5 Behavioural evaluation

A battery of behavioural tests was employed to comprehensively evaluate the effects of MSG and the protective potential of the plant extract on neurobehavioral functions. The Y-maze test was used to assess spatial working memory and hippocampal-dependent learning. The Elevated Plus-Maze evaluated anxiety-like behaviour and exploratory tendencies, reflecting amygdala and prefrontal cortical functions. The Open Field Test further measured general locomotor activity and emotional reactivity, providing complementary data on anxiety and exploratory drive. The Wire Suspension Test assessed neuromuscular coordination and muscle strength, which are often affected in neurotoxic conditions. Together, these behavioural paradigms offer an integrated profile of cognitive, emotional, and motor functions, allowing for a multidimensional evaluation of the neuroprotective effects of the test extract.

#### 2.5.1 Y-maze test

The Y-maze test was used to assess spatial working memory based on spontaneous alternation behaviour. The apparatus consisted of three identical arms (10 cm wide, 25 cm long, and 15 cm high) positioned at 120° angles. Each mouse was placed at the center of the maze and allowed to freely explore all three arms for 8 min. Prior to each trial, the apparatus was cleaned with 70% ethanol to eliminate olfactory cues. Arm entry was considered when all four limbs of the animal were within an arm. The sequence and total number of arm entries were recorded. Spontaneous alternation was defined as consecutive entries into three different arms (e.g., ABC, BCA, or CAB), while repeated entries (e.g., ABA, BAB, or CAC) were not considered alternations. The percentage of spontaneous alternation was calculated using the following formula: Spontaneous alternation (%) = (Number of alternations / (Total number of arm entries − 2) × 100. This parameter reflects hippocampus-dependent spatial working memory, as rodents naturally prefer to explore a less recently visited arm (Žnidaršič et al., 2024).

#### 2.5.2 Elevated plus-maze

The elevated plus maze (EPM) test was used to assess anxiety-like and exploratory behaviors in mice. The apparatus consisted of two open arms and two closed arms (enclosed by walls), arranged in a plus configuration and elevated above the floor. On day 10, one hour after treatment administration, each mouse was placed at the center of the maze facing an open arm and allowed to explore freely for 5 min. Prior to each trial, the apparatus was cleaned with 70% ethanol to eliminate olfactory cues. An arm entry was defined as the placement of all four paws into an arm. Behavioral parameters recorded included the number of entries and the time spent in open and closed arms. Additionally, ethological behaviors such as head dipping and rearing were also monitored as indicators of exploratory activity. Increased time spent and entries into the open arms are interpreted as reduced anxiety-like behavior, whereas preference for closed arms reflects higher anxiety levels. This test is widely used as a validated model of anxiety in rodents (Thurman et al., 2022).

#### 2.5.3 Open-field test (OFT)

It was done on a square arena (40 cm × 40 cm × 20 cm). The bottom of the arena was divided into sixteen squares (10 cm × 10 cm). Each animal was placed in a certain corner of the arena at the beginning of testing, and its behaviour recorded over 5 min (Zorkina et al., 2019). The parameters that were measured and recorded include: arena centre crossing frequency, time spent in the central square, total distance travelled, number of stretch attend postures, total squares crossed, rearing frequency, the total time of freezing (s), the grooming total time (s), and grooming frequency. The arena was cleaned after each animal trial with water and disinfectant to avoid phenomenon of place preference.

#### 2.5.4 The wire suspension test

Grip strength was assessed using the wire-suspension test (Brooks and Dunnett, 2019). Two poles were pinned 30 cm apart, and a taught wire was fastened to the poles at a height of 30 cm above the ground to form the contraption. The floor below the wire was filled with saw dust, in order to avoid animals from picking up injuries when they fall from the wire. Mice were held adjacent to the wire, and then released by the experimenter when holding the wire with both forepaws. The time each mouse took to fall from the wire was measured using stop watch and recorded. When a mouse successfully moved from one end of the wire (from one pole) to the other end of the wire without falling, then a maximum score of 60 s was given.

### 2.6 Sacrifice, collection of blood and brains

At the conclusion of the behavioural studies, animals from all experimental groups were euthanized under deep anaesthesia. Anaesthesia was induced via a single intraperitoneal injection of diazepam (10 mg/kg; purchased from Alembic Pharmaceuticals Limited, Karakhadi, Gujarat, India) and ketamine (50 mg/kg; purchased from Hikma Pharmaceuticals USA Inc., Berkeley Heights, New Jersey, USA). Adequate anaesthesia was confirmed by the absence of reflex responses before proceeding. Euthanasia was performed humanely through cardiac puncture while animals remained under deep anaesthetics conditions, in accordance with established guidelines for humane endpoints. Following euthanasia, the skulls were dissected to collect the brains, which were weighed using an electronic balance. Each brain was then divided: one portion was wrapped in aluminium foil, labelled, and stored at 2–4 °C for subsequent homogenate preparation, while the remaining portion was fixed in 10% (v/v) formalin (aqueous formaldehyde solution purchased from BC‑KC Formalin Ltd., Kazincbarcika, Hungary) for histological analysis.

### 2.7 Preparation of brain homogenate

Brain homogenates were prepared by homogenising the tissues in phosphate buffer (pH 7.4, 50 mM). Samples intended for biochemical analysis were centrifuged, and the resulting supernatants were stored at −20 °C.

### 2.8 Evaluation of biochemical parameters

#### 2.8.1 Evaluation of brain oxidative stress parameters

• Glutathione (GSH)
  Reduced glutathione (GSH) concentrations were determined spectrophotometrically using Ellman’s reagent (DTNB) as described by Ellman, 1959, with minor modifications. A volume (20 μl) of brain homogenates was introduced into test tubes, followed by 3 mL of Ellman's reagent (acid- 2,2-dithio-5,5′-dibenzoic acid). After homogenization, the tubes were incubated at room temperature for 1 hr. In the blank, the brain homogenates were replaced by 20 μl of 1 M phosphate buffer, pH 7.2. Their ODs read at 412 nm against the blank. The amount of cellular glutathione was express in micromole/mL according to the following formula:
  OD = Optical density
  **Glutathione = (OD + 0.0003)/ 0.3923** 0.3923 = Extinction coefficient
  0.0003 = Total quantity of brain homogenate
• Thiobarbituric acid reactive species(TBARS)
  Lipid peroxidation was assessed by quantifying thiobarbituric acid reactive substances (TBARS), primarily malondialdehyde (MDA), using a spectrophotometric method detailed by Draper, Hadley., 1990. A volume of 0.5 mL brain homogenate for each mice samples were introduced into individual test tubes followed by 0.5 mL of phosphate buffer pH 7.4, 50 mM and 1 mL of phosphate buffer pH 7.4, 50 mM was introduced into the blank test tube. 0.5 mL of 20% TCA (for protein precipitation) and 1 mL of 0.67% TBA were successively added to all the tubes (test and blank). The tubes were incubated at 90°C for 10 mins in the water bath after which all the tubes were cooled rapidly in ice. The content of each tube was transferred into a centrifugal tube and centrifuged at 3000 rpm for 15 min at 4°C. The supernatants were collected and their ODs read at 530 nm against the blank. The amount of MDA was calculated (F7).
  $$(F7)\;[\mathrm{MDA}](\mathrm{µmol}/L)\;=\;\frac{\Delta \mathrm{Abs}}{\varepsilon \times L\times m}$$
• Nitric oxide
  Nitric oxide (NO) levels were indirectly assessed by measuring its stable end products, nitrate (NO₃⁻) and nitrite (NO₂⁻), using a spectrophotometric assay, using Griess reagent. Brain homogenate (750) µL sample was added with 250 µL of Griess reagent and reaction mixture was Incubate for about 5–10 min at room temperature and protects it from light, the optical density was measured at 550 nm. Calculations were done after generating a standard curve from sodium nitrite in the same buffer as used for preparation of standard curve, and the amount of nitrite expressed as nmol/g tissue (Shinn, 1941).
• Superoxide dismutase (SOD)

Superoxide dismutase (SOD) activity was determined spectrophotometrically using a modified pyrogallol autoxidation inhibition method as originally described by Misra and Fridovich, 1972, with minor modifications. A volume (1600 μL) of carbonate buffer (50 mM, pH 10.2) was introduced into the cuvette, followed by 200 μL of brain homogenates. The reaction was initiated by adding 200 μL of adrenaline (0.6 mg/L) to all tubes, and after homogenization, the ODs were read at 480 nm. One unit of SOD is defined as the amount of SOD needed to inhibit 50% of oxidation of adrenaline to adrenochrome for one min. The percentage inhibition (% I) was equally calculated as follows (F8):

**(F**_**8**_**)** %I = (OD_sample_/OD_blank_) × 100

where OD is the optical density

$$\%\mathrm{of}\;\mathrm{inhibition}=\;100-\frac{\Delta \mathrm{Abs}\;\mathrm{test}}{\Delta \mathrm{Abs}\;\mathrm{blank}}\;x100$$

Where ΔAbs (variation of the different absorbance) = OD_80 s_ - OD_20 s_. If one unit of SOD (1U/mg tissue) induced 50% of inhibition, therefore n unit will induce X% of inhibition [15].

$$N=\left(X\%\right)x\frac{1}{50\%}$$

#### 2.8.2 Evaluation of acetylcholinesterase activity

Acetylcholinesterase (AChE) activity was determined spectrophotometrically using a modified Ellman, 1959 method, with methodological aspects optimized and discussed by Ellman et al., 1961. Tissue homogenates were prepared in 0.1 M phosphate buffer (pH 7.4) and centrifuged at 10,000 x g for 15 min (4°C)]. Samples were diluted in 0.1 M sodium phosphate buffer (pH 7.4). The reaction mixture (total volume of 1.0 mL) contained 0.1 M sodium phosphate buffer (pH 7.4), 0.3 mM 5,5′-dithiobis-(2-nitrobenzoic acid) (DTNB), and the diluted sample. After a 5-minute pre-incubation at e.g., 25°C, the reaction was initiated by the addition of acetylthiocholine iodide (ATCI) to a final concentration of 1.5 mM. The rate of 2-nitro-5-thiobenzoate (TNB) formation, reflecting AChE activity, was monitored as the increase in absorbance at 412 nm kinetically for 3 min using a spectrophotometer.

### 2.9 Histopathological examination

A portion of the brain was removed, preserved in labelled container containing 10% neutral buffered formalin, and then subsequently dehydrated using increasing concentrations of ethanol (70, 80, 90, 95 and 100%). Following dehydration, the samples were cleared with 2 changes of xylene. Followed by samples impregnation with 2 changes of molten paraffin wax, then embedded and blocked out. Sections of 5 μm were obtained using a rotary microtome and placed on glass slides. The tissue sections on the slides were stained using hematoxylin and eosin (H and E) staining according to Kada et al., 2021. Stained areas of control and treated rats were view under the light; OLYMPUS JAPAN at G40X, 100X and 200X. for histopathological changes.

### 2.10 Statistical analysis

Statistical analysis was performed using one-way analysis of variance (ANOVA), followed by the student–Newman–Keuls (SNK) post hoc test to determine significant differences between groups. Results are expressed as mean ± standard error of the mean (SEM). Differences were considered statistically significant at *p* < 0.05.

## 3 Results

### 3.1 Effect of *T. stapfiana* extract of spontaneous alternation in the Y-maze

**Fig. 1:**
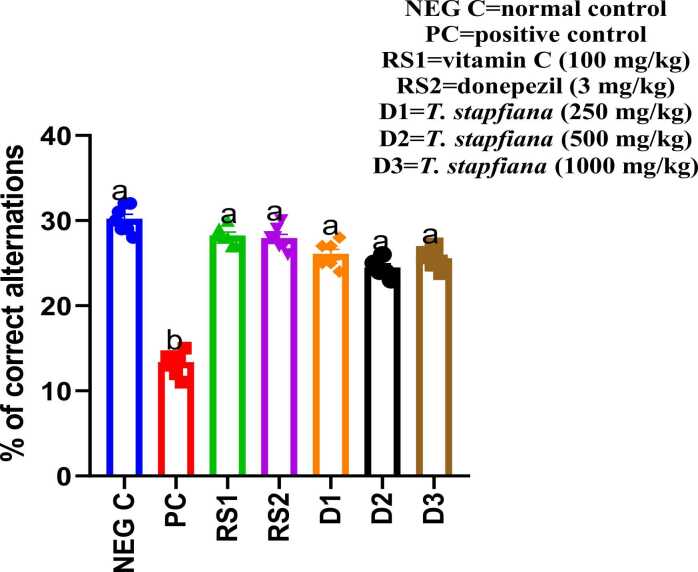
*T. stapfiana* extract on the percentage of correct spontaneous alternation in the Y-maze test. Each bar represents mean ± SEM, with seven mice per group. Significant differences exist between values that do not share a common letter (*P* < 0.05, Student–Newman–Keuls. NEG C= negative control, PC = positive control, RS1 =reference standard 1, RS2 =reference standard 2.

As illustrated in Fig. 1, monosodium glutamate (MSG) administration significantly (*p* < 0.05) impaired special working memory, decreasing the rate of spontaneous alternation from 31% in the negative control group to 11% in the MSG-treated group. In contrast, treatment with *T. stapfiana* (250, 500, and 1000 mg/kg), along with the reference drugs donepezil and vitamin C, significantly (*p* < 0.05) increased the rate of spontaneous alternation compared to the positive control group.

### 3.2 Effect of *T. stapfiana* extract of behavioural parameters in the elevated plus maze test

MSG toxicity induced anxiety-like behaviours in the elevated plus maze (EMP), as shown in Table 1. The number of entries into the open arms and the time spent in the open arms were significantly decreased in the MSG-treated mice (entries: 3.2 ± 0.58; time: 23 ± 0.89 s) compared to the negative control group (entries: 13.4 ± 0.93; time: 98.2 ± 0.8 s). Conversely, MSG toxicity significantly increased both the number of entries into the closed arms (26.6 ± 0.93 s) and the time spent in closed arms (44.3 ± 0.67 s) compared to the negative control group (entries: 9.8 ± 0.73; time: 11.8 ± 0.86 s). Donepezil, Vitamin C, and *T. stapfiana* extract (250, 500 and 1000 mg/kg) all effectively reverted these MSG-induced changes, increasing open arm exploration and decreasing closed arm exploration significantly (p < 0.05) compared to the MSG-treated group

**Table 1:** Results of the elevated plus maze test.

Parameters	Open arm entries	Close arm entries	Open arm duration (s)	Close arm duration (s)	Centre square entries	Centre square duration (s)
Treatments						
Negative control	13.4 ± 0.93^a^	9.8 ± 0.73^a^	98.2 ± 0.8^a^	11.8 ± 0.86^a^	17.6 ± 0.93^a^	108.4±1.18^a^
Positive control	3.2 ± 0.58^b^	26.6 ± 0.93^b^	23 ± 0.89^b^	44.3 ± 0.67^b^	5.8 ± 0.37^b^	43.25±0.97^b^
Vitamin C (100 mg/kg)	10.2 ± 0.66^a^	9 ± 0.89^a^	97 ± 1.14^a^	12.4 ± 0.81^a^	16.4 ± 0.51^a^	96.8±1.59^a^
Donepezil (3 mg/kg)	12.6 ± 1.03^a^	10.6 ± 0.51^a^	85.5 ± 1.69^a^	12.8 ± 0.37^a^	16 ± 0.71^a^	95.2 ± 0.86^a^
*T. stapfiana* (250 mg/kg)	11.6 ± 0.51^a^	10 ± 0.89^a^	97 ± 1.14^a^	11.6 ± 0.93^a^	15.4 ± 0.51^a^	108.8±1.16^a^
*T. stapfiana* (500 mg/kg)	10.8 ± 0.58^a^	10.4 ± 0.87^a^	83 ± 0.84^a^	14.8 ± 0.73^a^	12 ± 0.84^a^	94.2±1.17^a^
*T. stapfiana* (1000 mg/kg)	10.2 ± 0.58^a^	11 ± 0.45^a^	82 ± 1.05^a^	15.2 ± 0.86^a^	11.8 ± 0.86^a^	90.2±1.17^a^

values represent means ± standard error of means (SEM), n = 7 mice per group. Significant differences exist between values that do not share a common letter (P < 0.05, Student–Newman–Keuls).

### 3.3 Effects of *T. stapfiana* extract on locomotion, anxiety and exploratory behaviour of MSG-treated mice placed in open field

In the open field test (OFT), MSG administration also significantly (p < 0.05) altered exploratory and anxiety-related behaviours (Table 2). MSG-treated mice showed reduced locomotion, evidenced by significant decrease in total distance travelled (738±175.33 cm) and total line crossings (80.6 ± 19.68) compared to negative control (distance travelled: 1546±270.47 cm; total line crossings: 154.6±27.05). Exploratory behaviour in the central square was similarly impaired, with fewer entries (2.25±0.48) and shorter duration (2.25±0.48) in MSG treated mice compared to normal controls (entries:7.8±1.62; duration: 12.6±2.09). Moreover, MSG significantly increased anxiety, as indicated by an elevated rearing frequency (15 ± 1.38) compared to the negative control group (2.6 ± 0.24). Donepezil, vitamin C and *T. stapfiana* extract (250, 500, and 1000 mg/kg) effectively counteracted these MSG-induced changes, significantly (p < 0.05) increasing locomotion parameters (total distance travelled, total line crossings, central square entries and central square duration) and decreasing rearing frequency compared to the MSG-treated group.

**Table 2:** Results of the open field test.

Parameters	Distance travelled (cm)	Line crossed (cm)	Centre square entries (s)	Time in centre square (s)	Rearing without the wall	Rearing with aid of the wall	Grooming
Treatments							
Negative control	1546± 270.47^a^	154.6±27.05^a^	7.8±1.62^a^	12.6±2.09^a^	7.8±2.01^a^	2.6±0.24^a^	2.4±0.68^a^
Positive control	738±175.33^b^	80.6±19.68^b^	2.25±0.48^b^	2.25±0.48^b^	1.6±0.24^b^	15±1.38^b^	3.4±0.60^a^
Vitamin C (100 mg/kg)	1618±127.22^a^	141.4±13.34^a^	6.8±1.59^a^	10.6±1.08^a^	5.2±1.56^a^	2.8±0.37^a^	3±0.63^a^
Donepezil (3 mg/kg)	1460±100.95^a^	136.4±11.74^a^	7.4±1.08^a^	10.4±1.72^a^	6.8±1.88^a^	4±0.71^a^	3.2±0.73^a^
*T. stapfiana* (250 mg/kg)	1448±124.31^a^	144.8±12.43^a^	7.6±0.75^a^	11.8±1.50^a^	7.6±1.94^a^	2.6±0.24^a^	3.2±0.58^a^
*T. stapfiana* (500 mg/kg)	1162±127.18^a^	116±12.79^a^	5±1.38^c^	10.6±1.08^a^	6±1.67^a^	4.2±0.86^a^	2.6±0.60^a^
*T. stapfiana* (1000 mg/kg)	1416±53.91^a^	132.8±5.05^a^	4±0.71^c^	9.8±2.62^a^	5.8±1.80^a^	4.4±0.75^a^	2.2±0.49^a^

values represent means ± standard error of means (SEM), n = 7 mice per group. Significant differences exist between values that do not share a common letter (P < 0.05, Student–Newman–Keuls).

### 3.4 Effect of *T. stapfiana* extract on grip strength of MSG-treated mice in the wire suspension test

**Fig. 2:**
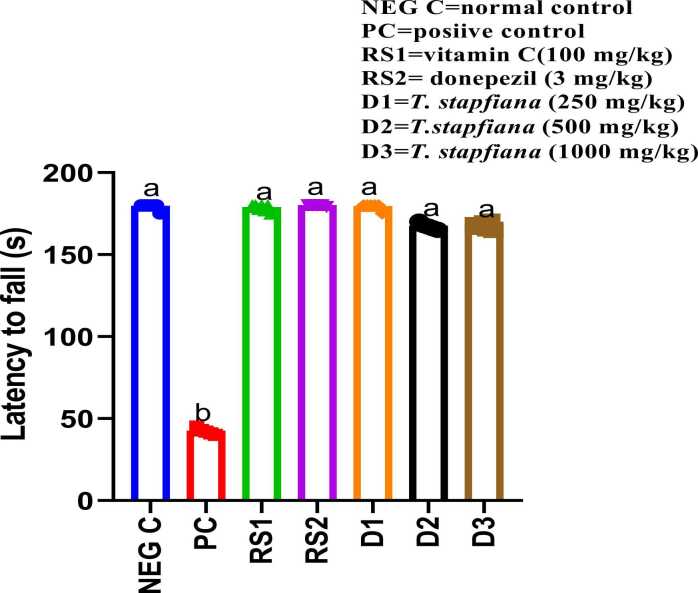
*T. stapfiana* extract on grip strength ability of mice. Each bar represents mean ± SEM, with seven mice per group. Significant differences exist between values that do not share a common letter (*P* < 0.05, Student–Newman–Keuls. NEG C= negative control, PC = positive control, RS1 =reference standard 1, RS2 =reference standard 2.

The hanging wire test (Fig. 2) revealed that MSG significantly impaired motor coordination and muscle strength, as indicated by a decreased latency to fall from 180 s in the negative control group to 40.6 s in the MSG treated group. All doses of *T. stapfiana* extract (250, 500, and 1000 mg/kg) effectively reversed this impairment, increasing latency to fall to 178, 160, and 150 s, respectively. Similarly, the reference drugs donepezil (177.6 s) and vitamin C (178 s) also restored latency to fall near normal levels.

### 3.5 Effects of the aqueous extract of *T. stapfiana* and MSG on acetylcholinesterase activity in brain tissue homogenate

**Fig. 3:**
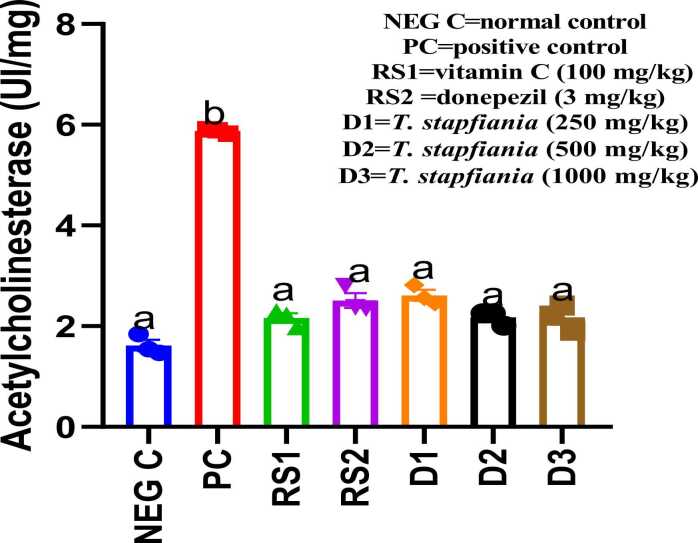
Effect of *T. stapfiana* extract on acetylcholinesterase activity of mice. Each bar represents mean ± SEM, with seven mice per group. Significant differences exist between values that do not share a common letter (*P* < 0.05, Student–Newman–Keuls. NEG C= negative control, PC = positive control, RS1 =reference standard 1, RS2 =reference standard 2.

As presented in Fig. 3, MSG administration significantly (*p<*0.05) altered brain acetylcholinesterase (AChE) activity. AChE activity was increased from 1.84 units in the negative control to 5.89 units in the positive control. This MSG-induced increase in AChE activity was significantly reduced by treatment with donepezil, vitamin C, and all doses of *T. stapfiana* extract (250. 500, and 1000 mg/kg) which decreases the activity to 1.98 U, 3.35 U, and 3.46 U respectively

### 3.6 Brain oxidative stress parameters

**Fig. 4:**
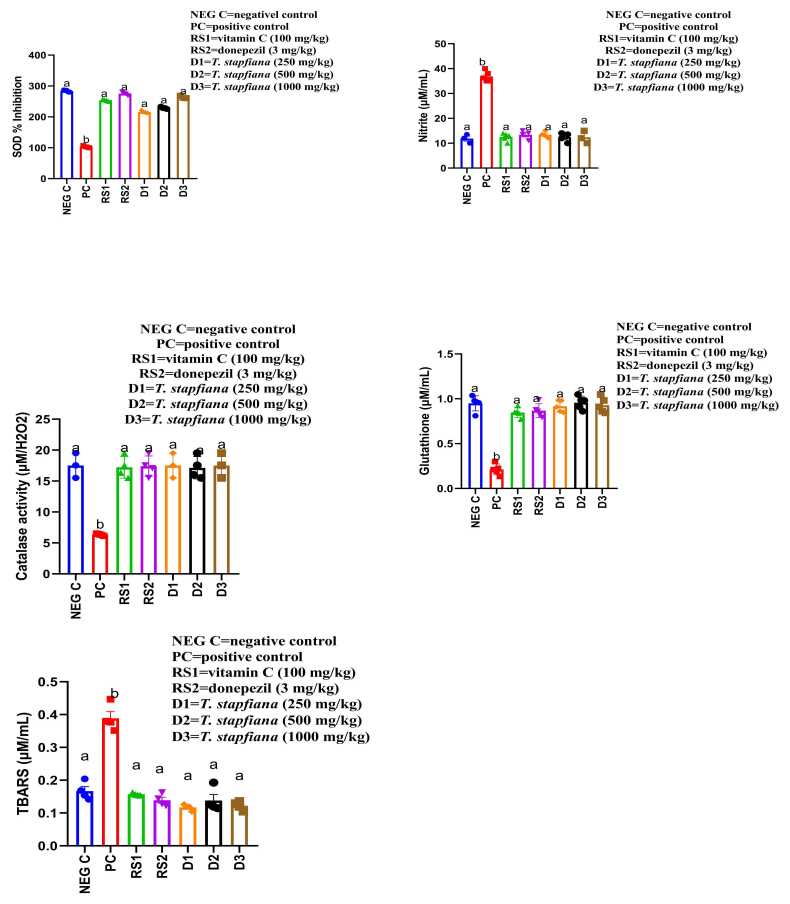
Effect of *T. stapfiana* extract on brain oxidative stress parameters of mice. Each bar represents mean ± SEM, with seven mice per group. Significant differences exist between values that do not share a common letter (*P* < 0.05, Student–Newman–Keuls. NEG C= negative control, PC = positive control, RS1 =reference standard 1, RS2 =reference standard 2.

As illustrated in Fig. 4, MSG induced significant oxidative stress in brain tissues, which was ameliorated by *T. stapfiana* extract treatment. In the positive control group (MSG-treated group), brain glutathione (GSH) levels, catalase activities and superoxide dismutase (SOD) were significantly (P < 0.05) reduced compared to the negative control group (normal rats that were not administered MSG). Similarly, superoxide dismutase (SOD) activity was drastically increased in negative control (normal rats that were not administered MSG). Concurrently, levels of thiobarbituric acid reactive substances (TBARS), an indicator of lipid peroxidation, and NO levels were significantly (P < 0.05) elevated in the MSG-treated group (positive control group) compared to negative controls (normal rats that were not administered MSG) (0.36 ± 0.0009 µmole/mL).

Treatment with *T. stapfiana* extract (all doses: 250, 500, and 1000 mg/kg), donepezil, and vitamin C significantly (P < 0.05) reversed these MSG-induced changes in oxidative stress biomarkers. GSH levels were restored by *T. stapfiana* doses with similar improvements seen for donepezil and vitamin C. SOD activity also increased back towards normal levels across all treatment groups. Correspondingly, TBARS levels and NO were significantly reduced in all treated groups, approaching negative control values.

### 3.7 Effect of the aqueous extract of *T. stapfiana* and MSG on histological examination of the cerebellum tissue in mice

**Fig. 5:**
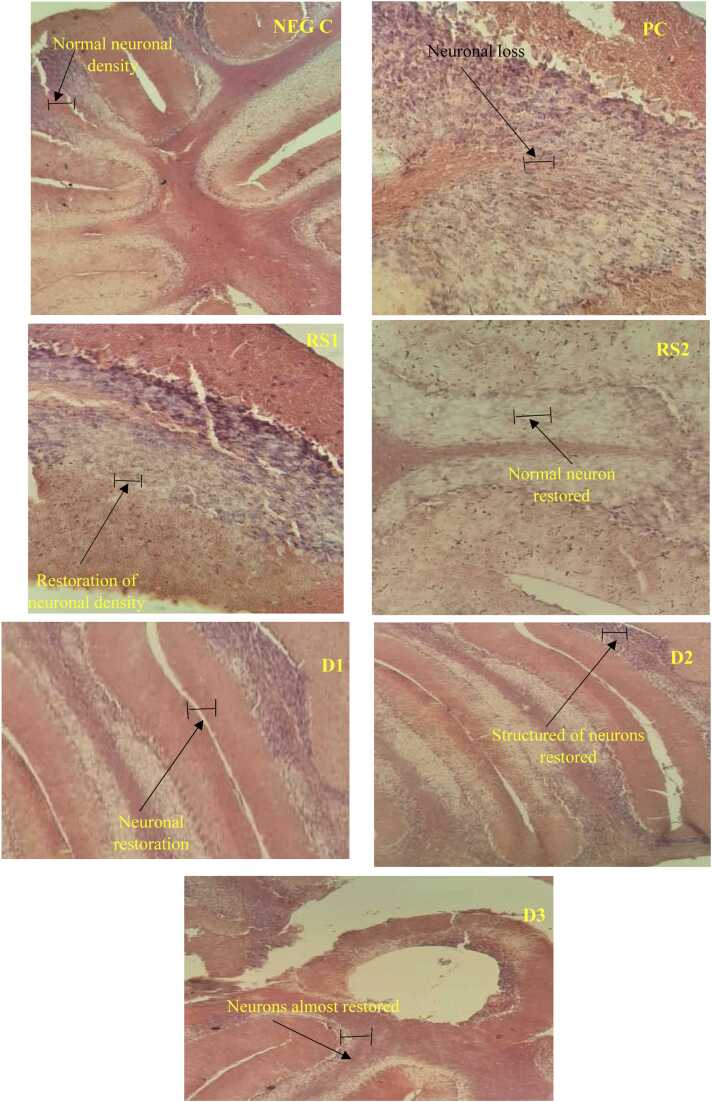
Microphotographs showing the effect of *T. stapfiana* extract on the general histology of the cerebellum of mice. NEG C: negative control group, PC: positive control group, D1: dose 1 (*T. stapfiana* 250 mg/kg), D2: Dose 2 (*T. stapfiana* 500 mg/kg), D3: Dose 3 (*T. stapfiana* 1000 mg/kg), RS1: reference standard 1 (Vitamin C 100 mg/kg), RS2: reference standard 2 (Donepezil 3 mg/kg). Hematoxylin & Eosin X100.

The cerebellar tissue of the brain was studied. Administrations of MSG for 10 days led to the reduction of the density of neurons in the cerebellar tissue. In the negative control group (NEG C), there is an orderly presentation of neuronal cells compared to the positive control group (PC) in cerebellar tissue. Therefore, there is little or no neurodegeneration in this group compared to the positive control group. In the positive control group (PC), there were devastating pathological alterations in the density of neurons in cerebellar tissue. Groups that were administered *T. stapfiana* extract showed restoration of neuronal cell density at all doses, with dose one (D1) showing the best protective activity when compared to the negative control group. This was equally seen with the positive control reference standard 1 (RS1) and reference standard 2 (RS2) groups which received vitamin C and donepezil respectively. Thus, the plant extract was capable of reversing the pathological consequences induced by this chemical substance in the brain of MSG-treated mice (Fig. 5).

## 4 Discussion

This study investigated the neuroprotective efficacy of the aqueous extract of *Tabernaemontana stapfiana* stem bark against monosodium glutamate (MSG)-induced neurotoxicity in mice. Although MSG is commonly consumed through the diet in humans, the intraperitoneal (*i.p*.) route was employed in this study to model neurotoxicity rather than to replicate dietary exposure. Oral administration is limited by first-pass metabolism and restricted systemic absorption of glutamate, making it less suitable for inducing consistent neurotoxic outcomes in experimental models. Consequently, the *i.p*. route is widely adopted in neurotoxicity studies as a validated approach to assess the direct neurotoxic potential of MSG.

Our findings demonstrate that monosodium glutamate (MSG) administration induces significant impairments in spatial memory, muscle strength, and increased anxiety-like behaviours, which are strongly associated with alterations in oxidative stress markers. These effects are closely linked to the dysregulation of key neurotransmitter systems, particularly glutamatergic and cholinergic pathways. MSG functions as an excitatory neurotransmitter in the central nervous system (Dingova et al., 2014), however, excessive glutamate exposure leads to excitotoxicity, characterized by prolonged neuronal depolarization, calcium overload, mitochondrial dysfunction, oxidative stress, and ultimately neuronal death (Dchanche et al., 2024). These pathological processes are well documented in the development of several neurodegenerative disorders, including Alzheimer’s, Parkinson’s, and Huntington’s diseases, as well as multiple sclerosis (Piniella and Zafra, 2023). In parallel, the cholinergic system plays a crucial role in cognitive and neuromuscular functions. Acetylcholine is essential for learning, memory, and synaptic transmission, and its activity is tightly regulated by acetylcholinesterase (AChE), the enzyme responsible for its degradation (Dchanche et al., 2024). MSG-induced oxidative stress has been shown to disrupt cholinergic neurotransmission by increasing AChE activity, thereby accelerating acetylcholine breakdown and impairing synaptic signaling. The elevated AChE activity observed in this study supports the occurrence of cholinergic dysfunction. Thus, the combined disruption of glutamatergic and cholinergic systems represents a central mechanism underlying MSG-induced neurotoxicity. The interplay between glutamate excitotoxicity and cholinergic impairment is increasingly recognized as a critical contributor to cognitive decline and neuronal damage.

The present study corroborates these mechanisms through the observed behavioural deficits. MSG-treated mice exhibited impaired spatial working memory, as evidenced by reduced spontaneous alternation behaviour in the Y-maze task, which depends on the integrity of the prefrontal cortex (Dingova et al., 2014). Additionally, MSG exposure induced heightened anxiety-like behaviour in the open field test, characterized by increased rearing frequency and stretch-attend postures, reflecting reduced exploratory activity and increased anxiety (Mattson, 2008). Furthermore, consistent with previous reports (Emégam et al., 2017), MSG significantly decreased muscle grip strength, highlighting its impact on neuromuscular function. Collectively, these findings underscore the widespread neurotoxic effects of MSG.

These behavioural alterations were accompanied by pronounced changes in oxidative stress parameters, further supporting the central role of oxidative stress in MSG-induced neurotoxicity. Specifically, MSG administration resulted in significant decreases in glutathione (GSH), superoxide dismutase (SOD), and catalase activities, alongside increased malondialdehyde (MDA) and nitric oxide (NO) levels in brain tissue. These findings are consistent with earlier reports indicating that MSG promotes oxidative stress and lipid peroxidation (Piniella and Zafra, 2023). Given that GSH, SOD, and catalase activities are essential components of the brain’s antioxidant defence system (Doukkli et al., 2016), their depletion, coupled with increased MDA levels and NO, reflects a severe imbalance between reactive oxygen species (ROS) production and antioxidant capacity. This redox imbalance contributes to neuronal dysfunction, membrane damage, and cell death. Importantly, pre-treatment with the aqueous extract of *Tabernaemontana stapfiana* effectively mitigated MSG-induced neurotoxicity at both behavioural and biochemical levels. The extract significantly improved spatial memory, reduced anxiety-like behaviours, and restored muscle strength. These functional improvements were accompanied by normalization of oxidative stress markers, including increased GSH, SOD, and catalase activities, and decreased TBARS and NO levels, indicating restoration of redox homeostasis. In addition to its antioxidant effects, *T. stapfiana* significantly attenuated the MSG-induced increase in AChE activity, suggesting preservation of acetylcholine levels and improved cholinergic neurotransmission. This effect is particularly relevant, as excessive AChE activity is associated with cognitive impairment and is a hallmark of neurodegenerative conditions such as Alzheimer’s disease. By inhibiting AChE activity, *T. stapfiana* may enhance synaptic availability of acetylcholine, thereby improving cognitive and neuromuscular functions. Moreover, by reducing oxidative stress, the extract may indirectly attenuate glutamate-induced excitotoxicity, further contributing to neuronal protection. The dual modulation of glutamatergic excitotoxicity and cholinergic dysfunction highlights a complementary neuroprotective mechanism underlying the effects of *T. stapfiana*. This mechanism may, in part, resemble that of Donepezil, a clinically used AChE inhibitor that enhances cholinergic transmission and improves cognitive performance. Overall, the ability of *T. stapfiana* to regulate oxidative stress, preserve cholinergic function, and mitigate excitotoxic damage supports its potential as a promising natural therapeutic candidate for the management of neurodegenerative disorders (Popa-Wagner et al., 2013).

Histopathological assessment in this study focused on the overall architecture of brain tissue, with particular attention to the Cerebellum, rather than exclusively targeting regions such as the hippocampus or cortex. The inclusion of the cerebellum was motivated by its well-documented vulnerability to excitotoxic and oxidative damage, especially in response to monosodium glutamate (MSG), due to the high sensitivity of Purkinje neurons to glutamate-mediated injury. Furthermore, the cerebellum plays a critical role in motor coordination and functional integration, making it a relevant structure for evaluating neurotoxicity and neuroprotection. This combined global and regionally relevant approach was adopted to provide a broad yet functionally meaningful view of MSG-induced neurotoxicity and the neuroprotective effects of the treatment. Similar generalized brain evaluations have been employed in previous research to assess neuronal degeneration, gliosis, and vacuolization as indicators of neurotoxic damage (Danysz and Parsons, 2012, Essawy et al., 2025). In addition, several studies have highlighted the cerebellum as a sensitive target in neurotoxicity models, particularly due to the selective susceptibility of Purkinje cells to oxidative stress and excitatory amino acids. The *T. stapfiana* extract, administered at different doses, prevented these histopathological alterations, confirming the presence of bioactive compounds with neuroprotective potential. Comparable findings were reported with *Sida acuta* leaf extract, which ameliorated MSG-induced microanatomical alterations in cerebellar Purkinje and pyramidal neurons (Ankul et al., 2023), and with Camellia sinensis extract, which preserved the structural integrity of brain tissue following MSG exposure (Owoeye O,Salami, 2024, Abdelhamid et al., 2024). Although region-specific histological analysis (e.g., hippocampus, cortex, and cerebellum) can yield more localized insights, the examination of whole-brain parenchyma, complemented by the inclusion of vulnerable regions such as the cerebellum, remains a valid and reproducible method for identifying generalized morphological changes associated with excitotoxic or oxidative injury. Future investigations could integrate more detailed region-specific quantification and immunohistochemical markers to further delineate the spatial distribution of neurotoxic and neuroprotective effects.

Although the present study employed an aqueous extract of *Tabernaemontana stapfiana*, the neuroprotective effects observed may be attributed to the presence of several bioactive phytochemical constituents reported in this genus. Previous phytochemical investigations of Tabernaemontana species have identified a rich composition of indole alkaloids (such as coronaridine, voacangine, and ibogaine-type compounds), flavonoids, phenolic compounds, and saponins, many of which exhibit potent antioxidant, anti-inflammatory, and neuroprotective properties. Indole alkaloids, in particular, are known to modulate neurotransmission, inhibit acetylcholinesterase activity, and protect neurons against excitotoxic and oxidative damage. Similarly, flavonoids and phenolic compounds contribute to free radical scavenging, enhancement of endogenous antioxidant defenses, and attenuation of neuroinflammation, all of which are critical mechanisms in the prevention of neurodegenerative processes. These classes of compounds have been widely associated with neuroprotective effects in experimental models of neurotoxicity and cognitive impairment. Therefore, it is plausible that the neuroprotective activity of the aqueous extract observed in this study results from the synergistic action of these phytoconstituents. However, further phytochemical characterization and isolation of specific active compounds are required to precisely identify the molecules responsible for these effects (Naidoo et al., 2021, Ngenteh et al., 2025).

## 5 Conclusion

The aqueous extract of *Tabernaemontana stapfiana* stem bark effectively protects against MSG-induced neurotoxicity in mice by improving cognitive and motor functions, restoring antioxidant balance, and preventing acetylcholinesterase overactivity. Histopathological analysis confirmed preservation of neuronal integrity. These findings suggest that *T. stapfiana* exerts multifaceted neuroprotective effects and may hold potential as a natural therapeutic agent for neurodegenerative and excitotoxic disorders.

## CRediT authorship contribution statement

**Salah Martin Anchang:** Writing – review & editing, Visualization, Validation, Supervision, Formal analysis, Data curation, Conceptualization. **Mumbi Laurentine Ngenteh:** Writing – review & editing, Visualization, Validation, Software, Methodology, Investigation, Data curation. **Kouémou Nadège Emégam:** Writing – original draft, Supervision, Software, Data curation, Conceptualization. **Kada Sanda Antoine:** Writing – original draft, Visualization, Validation, Supervision, Conceptualization. **Oumar Mahamat:** Supervision, Formal analysis, Data curation, Conceptualization. **Mboloh Patrick:** Writing – review & editing, Visualization, Validation, Methodology, Investigation.

## Declaration of Competing Interest

The authors declares that there is no conflict of interest regarding the publication of this article.

## Data Availability

The data will be available under request from the corresponding author.
